# A Different Way of Working: Embedding Clinical Psychology Placements in Third Sector Organisations Supporting People Experiencing Homelessness

**DOI:** 10.1111/hex.70334

**Published:** 2025-06-18

**Authors:** Hannah Frith, Mary John, Leah Sharkah, Jane Iles

**Affiliations:** ^1^ School of Psychology University of Surrey Guildford UK

**Keywords:** clinical psychology, homelessness, placements, third sector organisations, training

## Abstract

**Objectives:**

People experiencing homelessness experience high levels of trauma and psychological distress, but rarely access or engage with formal mental health services. The National Framework for Inclusion Health highlights collaboration between the NHS and third sector organisations as essential for reducing health inequalities. Providing clinical placements in third sector organisations for clinical psychology trainees may offer a valuable route to addressing this gap, whilst providing a beneficial learning experience.

**Methods:**

Qualitative semi‐structured interviews with clinical psychology trainees, staff in homeless settings and a clinical supervisor were used to explore perceptions of clinical placements and the benefits and challenges experienced by those involved in delivering these placements. Reflective thematic analysis was used to identify key themes in the perceptions of staff, supervisors and trainees.

**Results:**

Three key themes were developed. (1) *Bringing a psychological mindset* reflected the value that homelessness organisations placed on being helped to think about their client work through a psychological lens and extending this lens to also consider staff well‐being. (2) *Breaking barriers and building bridges* describes how trainees were positioned as brokers who could connect homelessness organisations to formal mental health systems and could advocate for homeless people within these systems in ways which may have long‐term effects. (3) *Working and learning differently* captures how these placements required trainees to work differently by crafting new roles and by adopting different working practices, including navigating complex issues around risk.

**Conclusions:**

Clinical psychology placements within homelessness organisations may help meet the objectives of the NHS National Framework for Inclusion Health by helping create PIEs in homelessness organisations, navigating connections between statutory and third sector organisations, and creating a skilful workforce adept at managing cultural mistrust.

## 1 Introduction

Mental health problems are prevalent among people experiencing homelessness (PEH) [1], yet they face many barriers to accessing healthcare, including: stigma; limited understanding of the link between homelessness, poor health, trauma and the criminal justice system among professionals; inflexible care delivery; and a lack of services for people with complex needs [2, 3]. Those struggling with substance misuse may find it particularly difficult to access care [4]. Practice guidance emphasises the need for NHS services to collaborate with homelessness organisations to reduce health inequalities and improve support for this population [5]. Community organisations supporting PEH face key challenges; they are often poorly/insecurely funded, a large proportion of their workforce consists of volunteer peer support workers with little formal training in mental health, and they have complex relationships to statutory funding and partnership working [6, 7, 8]. One attempt to bridge statutory services and community organisations involves co‐locating clinical staff in community settings, although this is not common [9].

## 2 Inclusion Health

The National Framework for Inclusion Health [5] outlines five principles underpinning an integrated approach to reducing health inequalities for PEH (and other marginalised groups):

1. Commit to action on inclusion health.
2. Understand the characteristics and needs of people in inclusion health groups.
3. Develop the workforce for inclusion health.
4. Deliver integrated and accessible services for inclusion health.
5. Demonstrate impact and improvement through action on inclusion health.

This paper speaks to the third principle—developing the workforce for inclusion health. Clinical psychologists are a core part of the National Health Service (NHS) workforce who support those with mental health difficulties. In addition to providing psychological interventions, they are expected to play a key leadership role by training and supervising professionals, influencing organisational policies and procedures, implementing improvements in service and patient care, and influencing national professional practice guidelines and policies [10, 11]. Recognising the potential for an upstream intervention into workforce development to support inclusion health, NHS England funded a pilot project across four universities in the South‐East of England to develop placements for clinical psychology trainees within homelessness organisations. We report the first phase evaluation from the implementation of this pilot at one university site.

## 3 Clinical Psychology Training

Doctoral training in clinical psychology in the United Kingdom is a competency‐based 3‐year programme funded by NHS England, combining academic work, research and clinical practice. Trainees are simultaneously NHS employees and students enrolled on a university programme accredited by the British Psychological Society. Successful completion of this course enables subsequent registration with the Health and Care Professions Council (HCPC), allowing individuals to practice as qualified clinical psychologists. Clinical practice placements in the NHS form a key part of the training, with 50% of trainees' time spent on placement across a variety of client groups and clinical settings. Like training in other healthcare professions, clinical placements offer opportunities to translate theory into practice and to develop/improve clinical skills [12]. Trainees highly value the learning gained from ‘doing and observing’ during clinical placements [13], where they also learn the implicit professional values in workplace practices and organisational cultures (or ‘hidden curriculum’) [14, 15].

Locating trainee clinical psychology placements within community organisations serving PEH offers the potential to develop the workforce for inclusion health by exposing trainees to a client population rarely encountered in statutory services and to workplaces with differing values and working practices. Moreover, organisations may benefit from access to otherwise unaffordable psychological expertise. Collectively, these placements have the potential to support action towards inclusion health and improve access to mental health provision for PEH. This evaluation aimed to identify the perceived effectiveness, benefits and limitations of clinical placements in homelessness organisations for clinical psychology trainees from multiple perspectives—trainee clinical psychologists, homeless organisations offering placements and clinical supervisors supporting trainees' practice.

## 4 Methods

### 4.1 Study Context

Five clinical psychology placements were sourced through a variety of outreach initiatives to build new partnerships with third sector organisations. In partnership with a local NHS Trust and another university clinical psychology training programme, we ran an online workshop to inform local authorities and community organisations about the qualifications and experience trainee clinical psychologists were likely to have, the work they are trained to complete, and how we anticipated trainees could contribute to the work of community organisations. Following this, organisations were invited to express interest in hosting a placement and were then contacted for a mutual assessment of the viability of hosting a placement within that organisation. In another geographical location, placements were sourced via local initiatives between the NHS Trust and local authorities. The five initial placements were based in organisations which varied in size and complexity, with some offering their own accommodation units and outreach services and some employing social workers, mental health workers and other professionals alongside peer workers. The organisations worked with varied PEH, including children and young people, rough sleepers, people in precarious housing, and those who were also experiencing substance misuse. Placements were 6 months in duration, with trainees being expected to achieve a minimum set of clinical competencies identified in advance, with opportunities for further competency development emerging during the placement. At the end of the placements, trainees, placement providers and supervisors were invited to participate in semi‐structured interviews to explore their experiences and evaluate the perceived effectiveness, benefits and limitations of these clinical placements. Invitations were sent via the administration team at the university to ensure independence, anonymity and privacy at the point of recruitment.

### 4.2 Data Collection

Semi‐structured interviews were conducted via video call and audio‐recorded by L.S., a research assistant who had no prior involvement in developing the placements or the training programme. The research team developed an interview topic guide tailored to the participant's role (i.e., supervisor, trainee and placement provider), but addressing similar areas: placement induction; learning attained; challenges experiences; comparison to other placements; suggested improvements; and outcomes which would be taken forward post‐placement. Automated transcripts were reviewed, corrected and anonymised by L.S. After each interview, L.S. had a debrief session with M.J. to reflect on the topics discussed in the interview and to consider whether the topic guide needed revision to draw out a richer understanding of participants' experiences.

### 4.3 Data Analysis

Data were analysed using reflexive thematic analysis (RTA [16]) to identify patterns in the meanings that participants made of their experience of doing/providing/supervising clinical placements and to draw on our own subjectivities as educators, practitioners and supervisors in interpreting these experiences. M.J., who brought a clinical/therapeutic lens, and H.F., who brought a qualitative research lens, coded the interview transcripts independently, which were drawn to different aspects of the data. M.J. was particularly drawn to perceptions of risk reflecting wider discussions in clinical settings, while H.F. was drawn to examples of learning being challenged and extended within the placements. Review of preliminary themes within the wider team ensured reflexive engagement with the developing coding and analysis. Developing themes were scrutinised for internal coherence and richness of data to support the theme. Data extracts are presented to illustrate the richness and complexity of the themes. To preserve anonymity, data extracts indicate a participant number and whether the speaker was a member of placement staff, a supervisor or a trainee clinical psychologist. Extracts have been edited to improve readability whilst preserving the original meaning by, for example, deleting pauses, hedges or incomplete words, inserting editorial notes in square brackets, or by removing words to join a thread of conversation together more concisely (indicated by […]).

### 4.4 Ethics

A favourable ethical opinion for the research was granted by the Research Ethics and Governance Committee at the University of Surrey (FMHS 22‐23 121 EGA). Due to the small number of trainees and organisations involved, it was not possible to ensure anonymity, and this was clearly communicated to participants.

## 5 Results

Seven people were interviewed: three placement staff based in community organisations, three clinical psychology trainees and one clinical psychologist who facilitated reflective practice sessions for the trainees. Two trainees and two placement staff did not respond to invitations to participate. Interviews ranged in length from 30 to 60 min.

**Figure 1:**
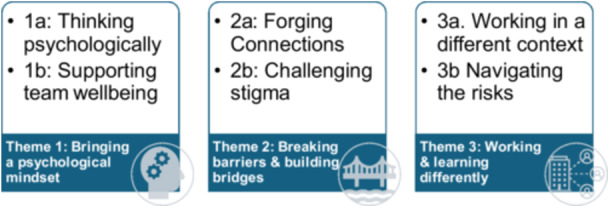
Summary of themes and sub‐themes.

Data analysis generated three themes with associated sub‐themes: (1) Bringing a psychological mindset; (2) Breaking barriers and building bridges; and (3) Working and learning differently (see Figure 1). Collectively, these themes describe the reciprocal benefits accrued by all parties when clinical psychology trainees are on placement within homelessness organisations, as well as some of the challenges experienced.

### 5.1 Theme 1: Bringing a Psychological Mindset

Service providers and trainees emphasised the valued work trainees did to upskill the team to ‘think psychologically’ about their clients and to recognise the impact of their work on the well‐being of the team.

*1a: Thinking psychologically*

A key identified benefit of locating clinical psychology trainees within community organisations was the mental health knowledge and experience that trainees brought into the service. Despite high levels of need, community organisations ‘*tend not to have clinicians in our services […] because we can't afford them, and they are rare’* (Staff, P6). Staff, including peer mentors (those with lived experience of homelessness), have many skills, but may have little or no psychological training. Trainees were valued for bringing ‘*new thinking’* (Staff, P3), ‘*clinical knowledge and understanding’* (Trainee, P2) and ‘*evidence about what works and what doesn't work’* (Staff, P6) into these services. This expertise was used to ‘*support the other professionals to have a more psychological understanding of what's going on’* (Trainee, P5), in ways which ‘*feeds into the development of the team’* (Trainee, P2) and enhances their ‘*knowledge and skills base’* (Staff, P6).

Upskilling staff teams was described as providing sustainable change within the organisation. To illustrate, one staff member described how group work initiated by the trainee had ‘*really strengthened our offer to clients’* and that the team retained ‘*the skills to run those groups as well, when the psychologists left’* (Staff, P6). For trainees, bringing a psychological mindset via education and training of the staff enabled them to develop skills and professional identities as leaders: ‘*I was very keen to expand on my leadership [skills] and I certainly see that as a big part of my role having had a lot of training in mental health and a lot more experience than a lot of the other staffing group here’* (Trainee, P5). Staff similarly recognised the value of the ‘*psychology leadership aspect’* of the placements for the organisation, including ‘*role modelling leadership within the teams, providing clinical advice, guidance and developing [the team]’* (Staff, P1).

*1b: Supporting team well‐being*

Bringing a psychological mindset was also evident in trainees' efforts to embed it into team practices that support staff well‐being. Given ‘*the level of trauma that we work with and the vicarious trauma that the staff are experiencing’* (Trainee, P2), creating spaces for staff to acknowledge the psychological impact of working with traumatised clients was highly valued. Introducing reflective practice sessions enhanced the skills of staff to set healthy boundaries with clients:

‘sometimes things pull on your heartstrings and I think one of the things that the psychologist has done is actually being able to help people understand why they feel like that and what their attachment issues are to the clients’.

(Staff, P6)

Combining space for reflection with theoretical knowledge (on attachment) led to new insights about the emotional dynamics between clients and staff. Similarly, introducing team formulation sessions afforded insight into clients alongside supporting staff development and well‐being:

‘[Trainee] did team formulation, kind of looking at cases and responses […] she leaves a legacy of reflecting on our work, reflecting on the impact on the staff teams, the trauma and transference of trauma […] sometimes it's hard to get a time to sit back and reflect ‘ok how has that affected us, how is that impacting on us’.

(Staff, P3)

Encouraging community organisations to adopt a psychological mindset and different working practices was sometimes difficult because ‘*it's not what they're used to and it's a different way of working, a different way of thinking’* (Trainee, P5). Trainees had to learn how to mobilise support to effect change by using ‘*relationships with the wider staff team to sort of allow me to develop the spaces’* (Trainee, P5). Developing these working processes allowed trainees to practice ‘*management and leadership’* (Staff, 1).

As such, the placements benefited staff well‐being while also enhancing the trainees' personal and professional development. Importantly, bringing a psychological mindset was perceived as having a sustainable benefit beyond the life of the placement. Staff noted that the trainee ‘*leaves something behind, and team formulation will stay and be something that we will work with’* (Staff, P3), they have ‘*have left a legacy through sort of upskilling staff’* (Staff, P6).

### 5.2 Theme 2: Breaking Down Barriers and Building Bridges

Placements provided opportunities to build connections between NHS and community‐based services, by challenging stigma, increasing understanding of PEH and community organisations, and reducing mistrust of psychologists/mental health services.

*2a: Forging connections*

While services welcomed the opportunity to benefit from psychological knowledge and expertise, they acknowledged some mistrust of psychological services/professionals arising from difficult personal experiences with statutory services among peer workers and the barriers to accessing care when advocating for mental health support for clients. A key benefit of locating (trainee) psychologists with community organisations was opportunities to develop mutual understanding and find ways to connect services. Trainees ‘*created a bit of a bridge between us and mental health services’* (Staff, P3). This included developing shared understandings by ‘*bust[ing] some of the myths that fit around what psychology is um, and what psychologists do’* (Staff, P6) and creating shared conceptual frameworks by ‘*starting to speak a similar language’* (Staff, P6) when communicating across service settings. With their ‘insider knowledge’ of the NHS, trainees were able to help organisations by ‘*navigating the system’* and ‘*creating those connections’* (Staff, P3) and by helping teams to ‘*gain a better understanding of what's happening for those* [NHS] *teams, the reasons for referral, the reasons why things can and can't be done’* (Staff, P1). As one placement provider explains, ‘*my staff come from an angle of being strong advocates for their clients’* who experience challenges when trying to access services for their clients, leading to ‘*a cynical view of other disciplines coming in’*. In contrast, she notes:

‘it's been a refreshing view to have [trainees] coming in and being such a positive influence and a positive part of the team. So, I think it's breaking those barriers down’.

(Staff, P6)

Trainees described working to build networks and systemic connections between services: ‘*I've kind of met with other services and try to link in the NHS, and offer training to staff and presentations to services just to try and pull together the networks […] I've been trying, whilst I'm outside of the NHS, to build that in’* (Trainee, P2). For trainees, developing a better understanding about how community organisations operate, including ‘*learning what is offered within a homelessness service’* (Trainee, P2), gaining ‘*understanding of who the services are, where they are, who they work with’* (Trainee, P5) and ‘*the challenges that staff face’* (Trainee, P4), left them feeling better prepared to connect with PEH and community services post‐qualification. Trainees also felt more aware of the need for integrated working in their role in developing partnerships with community organisations:

‘[post‐qualification] I would want to seek out opportunities where I can develop links with other services which if I hadn't had this placement, I don't think I would have really gone for. This placement has really underscored the importance of cross agency working, formulating and how much more efficient services can be when they work together’.

(Trainee, P2)

*2b: Challenging stigma*

These placements exposed trainees ‘*to a community which is not one that normally would be accessible’* within NHS‐based placements (Staff, P1). Through these placements, staff believe that trainees developed a deeper understanding of the stigma faced by PEH and the difficulties they experience in accessing psychological services:

‘I think she probably saw for the first time the reality of how hard it is for clients to access clinical services or mental health services generally’.

(Staff, P3)

They expected trainees to gain better insight into why clients may be ‘*disorganised or difficult to engage at times’* and to understand how ‘*the systems that are in place do not support people's ability to […] be able to access those services’* (Staff, P1). Trainees reflected on being exposed to a contrasting set of values and practices in community organisations, which they felt were more inclusive and person‐centred for PEH. They described a ‘*cycle of exclusion’* in NHS services where ‘*we wonder why people aren't getting better—it's because they are finding it really difficult, there's so many barriers for people to get help’* in contrast to homelessness services:

‘We work with people who are homeless, who are at risk of homelessness, you know, there's a lot of sort of prevention aspect to it as well. Um and you know we can be so flexible in the way that we try and engage people and try and work with them’.

(Trainee, P5)

Consequently, trainees reported feeling ‘*empowered’* to ‘*fight the stigma that there is against homelessness people’* (Trainee, P5) and ‘*feel more confident going into roles and um kind of advocating for clients’* to ensure they get the right support ‘*because I've kind of had to do that on this placement’* (Trainee, P2). Service providers hoped that placements would challenge trainees' perceptions of PEH and that, as *trainees*, they would take this learning forward to create systemic change post‐qualification:

‘…a big thing for us having placements is just about informing the professionals of the future and hope they don't stigmatise and treat homeless people with addictions and complex needs in the way the system can’.

(Staff, P3)

### 5.3 Theme 3: Working and Learning Differently

This theme captures a shared understanding that community organisations for PEH require a different way of working (with different norms, values and practices) than NHS placements and settings, which could be challenging for trainees to navigate, but also offered opportunities for creativity and growth.

*3a. Working in a different context*

Placements in NHS services are often well‐established, trainees have a recognised role within NHS services and hierarchies, and supervision is provided by a clinical psychologist familiar with the profession and training requirements. In community placements, the trainee may be the only psychologist in the service, organisations are typically smaller and less hierarchical, and trainees may work with clients and other professionals that they rarely encounter in NHS settings. Moreover, despite intensive prior planning (see [17]) as new placements in a different service context, inevitably teams were navigating how to implement these placements. Although these uncertainties meant trainees sometimes felt ‘*a bit like a Guinea pig’* or ‘*thrown in at the deep end’* (Trainee, P2), they also recognised that this context offered rich learning experiences.

In community organisations, working practices are attuned to trauma, complexity, chaotic lifestyles and poor access to basic needs. As one staff member notes, ‘*a lot of our clients fit within that fight and flight mode’* which means that ‘*everything else goes out the window in terms of how they prioritise their health’* so they ‘*don't turn up to appointments’.* Rather than withdrawing support after missed appointments, she goes on to explain that PEH require a different way of working:

‘Rather than them coming to you, you go to them or meeting them somewhere, which is like our day centres, which is a better environment because they feel safe and they're with trusted people rather than walking into a community health environment or to a hospital where they might feel completely overpowered and just run for the hills’.

(Staff, P6)

Trainees found it challenging to work with a client group who may ‘*have a lot of mistrust’* of services/professionals, feeling that they ‘*have let them down, excluded or rejected them in the past’* (Trainee, P5). Work has to be adapted to meet the needs of PEH:

‘You have to be very responsive. You have to look at shorter term interventions […] how to quickly assess, the shorter‐term assessments, how to engage people, how to motivate them […] It's a different way of working’.

(Staff, P1)

‘*The way of working in this system’* trainees learned ‘*is based on flexibility and focus on client need, as opposed to the service structures’* (Trainee, P4). Rather than following a structured therapeutic plan, trainees needed to ‘*do my work in a more casual way and sort of think creatively’* (Trainee, P4). Although sometimes challenging, trainees recognised that this also provided unique opportunities. As one noted ‘*I think the biggest skill or competency that I've kind of been developing from this is kind of leadership skills’* because there's ‘*great opportunities for leadership because there's so much scope to do anything you want on the placement and you just kind of have to go out there and get on with it’* (Trainee P2). The supervisor also reflected on this, observing that trainees were ‘*positioned in a slightly different way in terms of leadership’ due to being perceived as external to the team and there as ‘a consultant to that team as opposed to a member of that team’* (P7).

*3b Navigating challenges and risks*

While community organisations offer flexibility and creativity, the lack of infrastructure can be challenging. Trainees articulated a number of personal and professional risks associated with supporting PEH in these contexts. For example, developing initiatives can be exciting and rewarding, but can also be time‐consuming and exhausting: ‘*… setting up these processes, figuring out the role, meeting with teams, because you're new, it takes a lot of time to do those things’* (Trainee, P2). As this trainee goes on to observe, this kind of work can be invisible or ‘*hard to quantify’,* leaving trainees feeling that their achievements are not evident. Trainees also reflected on the impact of working in the fluid landscape of seeing clients in the community, without appointments or preparation time and with limited access to colleagues' support. Working in low‐resource settings was also challenging. Some trainees did not have an office and were having to work from their cars, which led to a sense of isolation and having to manage their own emotional toll of this and the work:

‘Um not having a base is terrible like I just can't wait to go back and have an office. I think it's really emotionally taxing when you go from client to client, and you don't have anywhere to like to turn off in between. Um so there's no space where I just get to be me, I always feel I have to be a psychologist’.

(Trainee, P4)

Having few opportunities to debrief and share the challenges of clinical practice, or no place ‘to turn off’ or ‘rest’, meant that trainees had to develop high levels of personal resourcefulness and resilience to manage this emotional burden. As the supervisor (P7) noted, on these placements, trainees ‘*need quite a high degree of autonomy because often there's not a psychology—your supervisor isn't on site and also because of the way that services are structured’*.

Finally, trainees drew attention to the increased physical risks associated with working with vulnerable/complex clients in their own community settings:

‘like the risks are way different cause I'm going out to do community work, the risks are I could be attacked if I'm with a client who has like a risk of drug deaths and stuff like that’.

(Trainee, P4)

Trainees reflected on working with complex clients with high levels of distress within organisations whose values and governance procedures were perceived as somewhat dissimilar to those in NHS services:

‘when you're working with the service that doesn't have all the like policies and procedures that the NHS has, that has loads of benefits, but it also makes it like more unsafe or more dangerous for people working there’.

(Trainee, P4)

Protocols for risk assessment within the homelessness sector tended to be more person‐centred and had a dynamic quality to their operationalisation rather than being governed by protocols and policies as is the case in NHS settings. Trainees were encouraged to detect potential new risks and to explore the client's willingness to engage with risk‐taking. Nonetheless, the rapidly changing context meant that decisions often had to be made rapidly, ‘*so it's a different way of working’* (Trainee, P4). In community contexts, an immediate response is enacted to enhance safety in that moment, as future engagement with the practitioner is more uncertain. Overall, despite these challenges, trainees felt that they had gained a ‘*really unique and incredible opportunity that I've learned so much from […] I will really clearly this remember this placement for a long time to come and I think that one of the most powerful elements was being situated outside of the NHS’* (Trainee, P2).

## 6 Discussion

This study explored the benefits and challenges of providing trainee clinical psychology placements in homelessness settings as a way of improving the provision of psychological services for PEH and developing a psychological workforce equipped to meet the challenges of working with marginalised groups. Drawing on the experiences of trainees, clinical supervisors and service managers in organisations supporting PEH, the results highlight how such placements can provide sustainable benefit to staff working in homelessness settings, as well as upskilling trainees to effectively engage marginalised people in services with a clear legacy left for all. We discuss the findings in relation to leadership for inclusion health and opportunities for transformative learning.

## 7 Leadership for Inclusion Health

Both trainees and staff identified that community placements offered important opportunities to shape the personal and professional values and skills of trainees, and by extension, the future generation of psychology leaders. Calls for better access to/experience of mental health services for marginalised groups have highlighted the need for more explicit teaching and training on leadership skills for clinical psychology trainees [18].

Participants identified placements within the community homelessness sector as providing non‐typical but valuable opportunities for trainees to develop and practice their leadership skills ‘on the job’ in busy, complex environments with staff teams who are under pressure and with clients facing multiple difficulties. The complexity of clients and community settings challenged trainees to develop leadership and supervision skills appropriate to their level of training, including developing reflective practice, integrating the evidence base into practice, and service improvement (BPS, 2010). These leadership skills include promoting psychological mindedness and skills in other health, educational and social care providers (BPS, 2024). Community organisations benefited from enhanced service provision; psychologically informed practice, staff training and skill development; and increased understanding of and networking with psychological services, in ways which were sustainable beyond the end of the placement. Psychologically informed environments (PIEs) where ‘staff are trained to develop an increased psychological understanding of the work that they do’ ([19], p. 52) are highly relevant to managing the trauma associated with experiencing homelessness and may offer a mechanism for reducing staff burnout [20]. The community organisations valued the trainees' sharing knowledge about specific mental health difficulties, developing a shared language to talk about these difficulties across services, and providing opportunities to reflect as a team about their own experiences of working with trauma and complexity (see also [21]). By bringing a psychological mindset, introducing mechanisms for supporting staff well‐being and finding ways to build bridges between statutory services and community organisations, our research demonstrates that the work trainee clinical psychology did on placement was able to make a sustainable contribution to developing a psychological mindedness within community organisations. Future research which empirically examines the longer‐term sustainability of these contributions, the impact of these on the psychological mindedness and well‐being of staff, and the impact on PEH who access these services, would establish the value of these initiatives for the organisations. Placement staff and the clinical supervisor emphasised how they hoped that interactions with PEH would lead to a better understanding of the challenges they experience in accessing mental health services and the stigma that they face. As future leaders, community organisations expected the trainees to take this awareness and understanding back into NHS services and advocate for PEH in the future. These hopes and expectations were confirmed by the trainees who reported a greater understanding of the stigma experienced by PEH and the importance of advocating for services which would meet the needs of this group.

## 8 Transformative Learning

We argue that providing placements in community organisations supporting PEH offered trainees opportunities for transformative learning. Transformative learning occurs when adults are faced with a disorientating dilemma which provokes critical self‐reflection, elaborating existing frames of reference or learning new ones, and transforming habits, values or points of view [22]. These placements offered a unique experience for trainees to adapt and develop ways of engaging marginalised people. They challenged trainees not only to develop new skills and to flexibly adapt their knowledge and skills to the context and the client group (meeting them where they are at), but also to reconsider their attitudes and beliefs about PEH and models of service delivery. Demanding and novel learning opportunities help people to learn independently and (in this case) adapt what they know to novel and complex environments and develop confidence and competence in new roles and relationships. As such, we argue that (at least for some) it provided opportunities to engage in personal transformative learning, which they anticipated would have long‐lasting consequences for their personal and professional development. This potential was recognised by supervisors and service managers for whom shaping the values, beliefs and knowledge of future clinical psychologists was an important motivator for participating in the scheme. Indeed, out of five trainees who undertook the initial placements, three have gone on to work with homelessness services post‐qualification. Transformative learning is widely discussed in other professional contexts (such as nursing, see [23]) but is rarely discussed in the context of clinical psychology training. Future research might examine more closely the potential of these placements for transformative learning and the longer‐term impact on professional development post‐qualification.

## 9 Strengths and Limitations

This novel pilot project study provided wide‐ranging insights from trainees, service managers and supervisors about the experience of providing trainee clinical psychology placements within community organisations supporting PEH and identified key areas for future research. Limitations include the small sample size and the failure to examine the direct impact of the placements on PEH themselves.

## 10 Conclusion

Locating clinical psychology trainee placements within community organisations which support PEH can play an important role in progressing the NHS drive for inclusion health. Such placements can be mutually beneficial for the organisations (in terms of staff development and enhanced service provision) and for trainees (in terms of opportunities for leadership and experience of working with complexity). In the longer term, upskilling trainee psychologists to become strong advocates for inclusion, who understand how to engage marginalised communities and forge connections between the NHS and community organisations, may create opportunities to move towards integrated care for marginalised populations.

## Author Contributions

**Jane Iles:** conceptualisation, funding acquisition, supervision, writing – review and editing, methodology. **Hannah Frith:** conceptualisation, formal analysis, writing – original draft, visualisation, validation, methodology. **Mary John:** conceptualisation, formal analysis, writing – original draft, validation, supervision, methodology. **Leah Sharkah:** investigation, data curation, project administration.

## Conflicts of Interest

The authors declare no conflicts of interest.

## Patient and Public Contribution

- Upskilling a psychologically informed workforce: By embedding psychologically informed practices, such as team formulation and reflective practice, and upskilling staff to develop a psychological mindedness, trainee clinical psychologists contribute towards enhanced service provision for people experiencing homelessness (PEH) and support staff well‐being.
- Transformative learning: Being exposed to the complexities of experiencing homelessness and providing services in fast‐moving, low‐resource organisations challenges trainee clinical psychologists to adapt their clinical skills to engage this population, develop new working practices, recognise the limitations of statutory provision, acknowledge stigma and learn to advocate for people experiencing stigma.
- Leadership for inclusion health: Developing clinical placements within community organisations supporting PEH fosters the leadership skills necessary to develop inclusive and sustainable mental health provision.
- The role of consultation with the homeless community: We listened to community organisations and peer workers to direct the focus of placement opportunities to account for the varying local needs of the homeless population.

## Data Availability

The data that support these findings of this study are available from the corresponding author upon reasonable request.
